# Stem cell‐derived mitochondria transplantation: A promising therapy for mitochondrial encephalomyopathy

**DOI:** 10.1111/cns.13618

**Published:** 2021-02-03

**Authors:** Kaiming Liu, Zhijian Zhou, Mengxiong Pan, Lining Zhang

**Affiliations:** ^1^ Department of Neurology The Second Affiliated Hospital Zhejiang University School of Medicine Hangzhou China; ^2^ Department of Neurology Shaoxing Hospital of Traditional Chinese Medicine Affiliated with Zhejiang Chinese Medical University Shaoxing China; ^3^ Department of Neurology First People's Hospital of Huzhou Huzhou China; ^4^ Shanghai Jiaotong University School of Medicine Shanghai China

**Keywords:** mitochondria, mitochondria quality control, mitochondrial dynamics, mitochondrial encephalomyopathy, stem cell, tunneling nanotube

## Abstract

Mitochondrial encephalomyopathies are disorders caused by mitochondrial and nuclear DNA mutations which affect the nervous and muscular systems. Current therapies for mitochondrial encephalomyopathies are inadequate and mostly palliative. However, stem cell‐derived mitochondria transplantation has been demonstrated to play an key part in metabolic rescue, which offers great promise for mitochondrial encephalomyopathies. Here, we summarize the present status of stem cell therapy for mitochondrial encephalomyopathy and discuss mitochondrial transfer routes and the protection mechanisms of stem cells. We also identify and summarize future perspectives and challenges for the treatment of these intractable disorders based on the concept of mitochondrial transfer from stem cells.

## Graphical abstract

This review is mainly about the present status of stem cell therapy for mitochondrial encephalomyopathy, the triggering mechanisms for mitochondrial release, pathways of mitochondrial delivery, and the protection mechanisms on recipient cells.

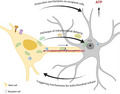

## 1 INTRODUCTION

Mitochondria are multifunctional cellular organelles that have a critical role in energy production via oxidative phosphorylation.^1^, ^2^ Mitochondria not only generate adenosine triphosphate (ATP, Table 1) during the oxidative phosphorylation (OXPHOS) process, but also contribute to many other processes, such as cell survival and autophagy.^3^ The OXPHOS complexes in mitochondria are dually encoded by the mitochondrial DNA (mtDNA) and the nuclear DNA (nDNA). Mutations in mtDNA or mitochondrial nDNA can cause fatal or severely debilitating disorders,^4^ such as mitochondrial encephalomyopathies, which occur in the neuromuscular system.

**TABLE 1:** The list of abbreviations in the paper

Abbreviations	
ATP	adenosine triphosphate
BNIP3	Bcl2 interacting protein 3
Cx43	connexin 43
Drp 1	dynamin‐related protein 1
EVs	extracellular vesicles
Fis1	fission 1 protein
IL	cytokines interleukin‐1
LC3	light chain 3
MERRF	myoclonic epilepsy with ragged red fibers
Mfn1	mitofusin 1
Miro	mitochondrial Rho‐GTPase
MNGIE	mitochondrial neurogastrointestinal encephalopathy
MSC	mesenchymal stem cell
mtDNA	mitochondrial DNA
nDNA	nuclear DNA
OPA1	optic atrophy 1
OXPHOS	oxidative phosphorylation
PINK1	putative kinase 1
TNFα	tumor necrosis factor alpha
TNT	tunneling nanotube
TP	thymidine phosphorylase
TRAK	trafficking kinesin protein

The traditional “mitochondrial cocktail” has little therapeutic effect on mitochondrial encephalomyopathies.^2^ Mitochondria dysfunction have unusual characteristics that may be treated from the cell level to the molecular level.^2^ Gene therapy prior to conception and reproductive technology to uncouple the inheritance of mtDNA from nDNA may offer possible solutions for mitochondrial encephalomyopathies. However, treatments for current mitochondrial patients still face numerous challenges. By understanding the molecular pathogenesis of mitochondrial diseases, it has been possible to develop some targeting therapy approaches, such as DNA manipulation and small‐molecule pharmaceuticals.^2^, ^5^, ^6^ However, the heterogeneity of mitochondrial encephalomyopathies and double‐membrane structure make these therapy approaches difficult to materialize.^2^, ^7^ The development of stem cell therapy may offer great promise for mitochondrial encephalomyopathies.^2^, ^8^, ^9^, ^10^, ^11^, ^12^, ^13^, ^14^, ^15^ The therapeutic mechanism includes paracrine cytokines, modulation of the immune system, and transdifferentiation effects.^2^, ^16^ Recently, stem cells have been found to donate healthy mitochondria to injured cells to rescue aerobic respiration and recover their metabolism capability. This is being considered as a new therapeutic strategy for tissue damage,^17^, ^18^ especially for mitochondrial diseases.^15^ Here, we summarize and discuss the current research on mitochondrial transfers and their protection mechanisms to provide an update on stem cell therapy targeting mitochondrial encephalomyopathy.

## 2 CLINICAL AND PRECLINICAL EVIDENCE FOR STEM CELL THERAPIES IN MITOCHONDRIAL ENCEPHALOMYOPATHY

More and more research evidence supports the effects of stem cell therapy in some neurological diseases with mitochondrial dysfunction.^19^ One of the most representative mitochondrial diseases that benefits from stem cells therapy is mitochondrial neurogastrointestinal encephalopathy (MNGIE), which is due to thymidine phosphorylase (TP) gene mutations, leading to secondary mitochondrial DNA damage.^20^ Stem cell therapies can recover TP enzyme function and improve the prognosis of MNGIE patients, which provides the initial evidence to support the effects of stem cell therapy in mitochondrial encephalomyopathy.^20^, ^21^, ^22^, ^23^ The relative lack of mtDNA‐based animal models limits stem cell research on mtDNA‐related mitochondrial encephalomyopathies.^24^ Encouragingly, some in vitro studies have demonstrated stem cells donate healthy mitochondria to replace dysfunctional mitochondria and recover energy metabolism in different types of recipient cells.^18^, ^36^ Mesenchymal stem cells (MSCs) are shown to transfer their own mitochondria into mtDNA‐depleted cells.^37^ Moreover, Wharton's jelly MSCs can also transfer mitochondria to stressed mitochondrial encephalopathy fibroblasts to eliminate mutation burden, rescue mitochondrial functions, and resist against apoptotic stress, which demonstrates the protective effects of stem cell‐derived mitochondria transplantation in an in vitro model of mitochondrial encephalomyopathy.^15^, ^38^ Recently, in an in vivo study, transplanted pluripotent stem cell‐derived MSCs can transfer their own mitochondria to recipient cells to protect against damaged retinal ganglion cell.^35^ These findings pave the way for clinical therapy study on mtDNA‐related mitochondrial encephalomyopathies through stem cell‐derived mitochondrial transplantation.

## 3 TRIGGERING MECHANISMS FOR MITOCHONDRIAL RELEASE

The transfer of stem cell mitochondria is a complex and intriguing phenomenon. The intercellular communication between recipient cells and stem cells may set up a specific "find‐me" and "rescue me" signal connection in the local injured regions.^18^ Mitochondrial damage appears to be the main trigger for release of the mitochondria.^39^ For the mitochondrial encephalomyopathies, injured mitochondrial components and other molecules are secreted to the periplasmic space as triggering signals by stressed cells.^40^, ^41^ Mahrouf‐Yorgov et al. found MSCs engulfed the mtDNA released by the co‐cultured cells with mitochondrial dysfunction. They reported there were subsequently increases in cytoprotective enzyme heme oxygenase‐1 expression that potentially enhanced the mitochondrial donation capability of MSCs.^42^ The loss of cytochrome c can trigger mitochondrial transport from stem cells to the injured cells.^43^ In addition, mitochondrial components and mtDNA also play a role in damage‐associated molecular patterns.^44^, ^45^, ^46^, ^47^, ^48^ The cytokines interleukin‐1 (IL‐1), IL‐4, IL‐10, and tumor necrosis factor alpha (TNFα) can be perceived by stem cells and can also act as triggering signals.^49^ Jiang et al. found that the high production of TNFα from the retinal ganglion cells results in mitochondrial release from stem cells.^35^ The translocation of p53 in neurotoxic recipient cells sends out a danger signal to donor MSCs to prompt healthy mitochondrial transfer.^50^ When stem cells receive these triggering signals from cells with mitochondrial dysfunction, the intrinsic mechanisms in stem cell begin to regulate the mitochondria transfer.^49^, ^50^, ^51^ The CD38/CADPR/Ca^2+^ pathway is also shown to mediate astrocytes to provide their mitochondria to the damaged neurons.^52^ It is necessary to explore whether the CD38/CADPR/Ca^2+^ pathway also works in stem cells. These findings suggest that stem cells might perceive some degree of metabolic dysfunction in adjacent cells with mitochondrial disorders and prepare to initiate mitochondrial transfer.

## 4 PATHWAYS OF MITOCHONDRIAL DELIVERY

**FIGURE 1:**
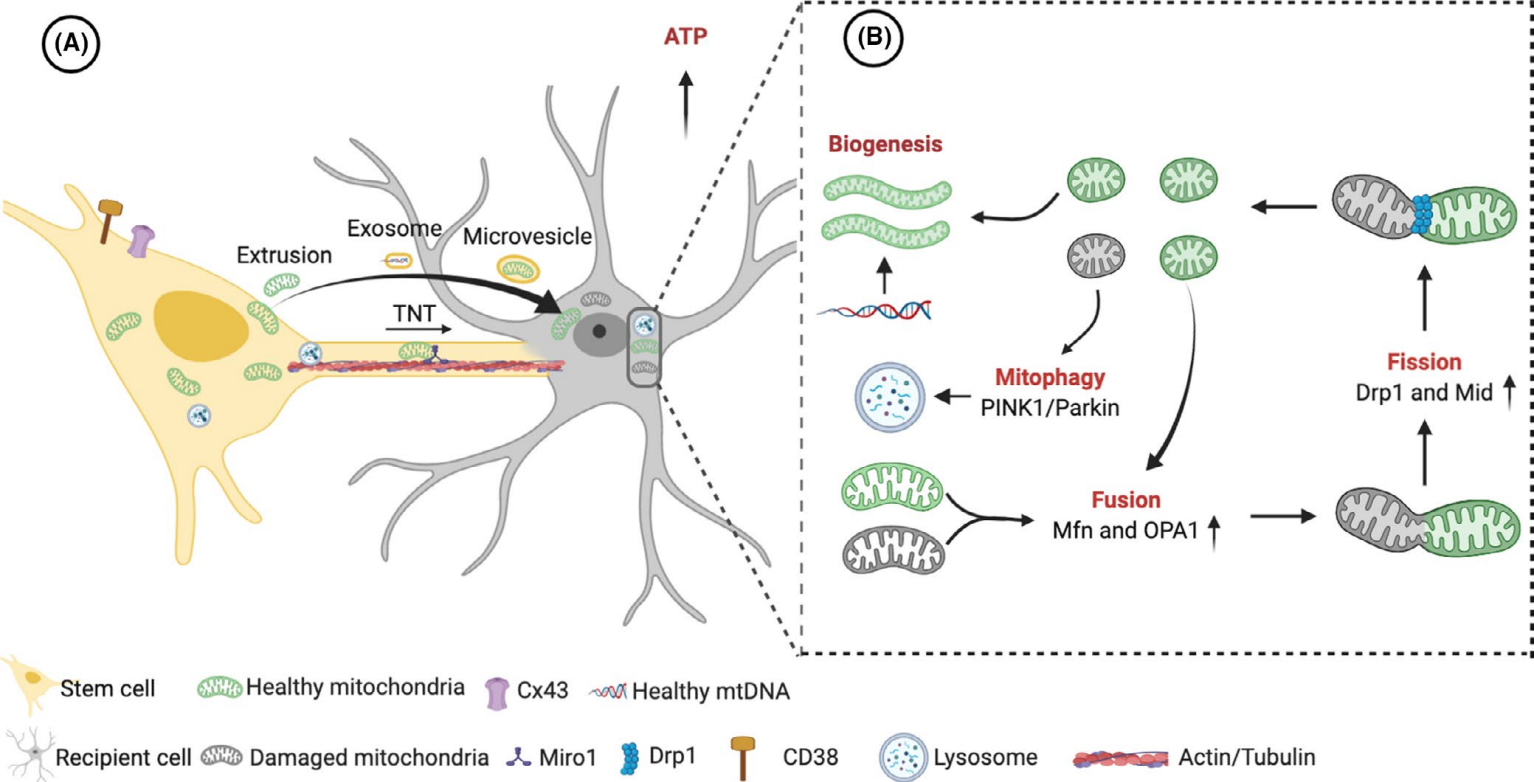
Transfer routes and protection mechanisms of stem cell‐derived mitochondria. (A) The routes of healthy mitochondria transfer from stem cells to recipient cells with dysfunctional mitochondria include TNT formation, release of extracellular microvesicles and mitochondrial extrusion. Exosomes might transfer organelle fragments (such as protein complexes of the mitochondrial electron transfer chain), mtDNA and ribosomes. The permanent cell fusion and formation of synkaryons are scarce in co‐culture conditions and in vivo, which are not drawn in the figure. (B) Stem cell‐derived mitochondria might rescue aerobic respiration and energy metabolism directly, regulate mitophagy and mitochondrial biogenesis, optimize mitochondrial fission and fusion, and decrease the mtDNA mutation load. Cx43, connexin 43; Drp 1, dynamin‐related protein 1; Miro1, Mitochondrial Rho‐GTPase 1; mtDNA, mitochondrial DNA; Mfn, mitofusin; Mid, mitochondrial dynamics protein; OPA1, optic atrophy 1; PINK1, putative kinase 1; TNT, Tunneling nanotube. (Figure Created with BioRender.com)

Several different routes have been found to participate in mitochondrial transmission from stem cells to recipient cells, which include tunneling nanotube (TNT) formation, release of extracellular microvesicles, cellular fusion, and mitochondrial extrusion (Figure 1A). The molecular mechanisms mediating different intercellular transmission routes are complex. A clear understanding of these routes and mechanisms will be a benefit to stem cell therapy in mitochondrial diseases.

### 4.1 Tunneling nanotubes

Tunneling nanotubes are actin‐based cytoplasmic extensions connecting cells as intercellular channels 50–1000 nm in diameter in a wide variety of cell types. Ramírez‐Weber initially described a kind of membrane nanotube when studying drosophila wing imaginal disks.^53^ The tunneling nanotubes were then defined by Rustom et al. in a rat PC12 cell‐human 293 cell co‐culture.^54^ As a new mechanism of intercellular communication, TNTs promote the exchange of signaling molecules and cellular components between cells such as Ca^2+^, nucleic acids, pathogens, organelles, and plasma membrane components, including mitochondria.^18^, ^54^, ^55^ A variety of motor proteins enable efficient transport of mitochondria between connected cells,^56^ like mitochondrial Rho‐GTPase 1 (Miro1) and Miro2,^57^, ^58^ KLF 5 kinesin motor protein,^59^ trafficking kinesin protein 1 (TRAK 1) and TRAK 2,^60^, ^61^ and Myo 19^62^ and Myo 10.^63^ MSCs have been shown to rescue damaged cells through TNT‐mediated mitochondrial transmission. MSCs detect a "find‐me" signal gradient, form membrane protrusions, and extend to create TNT with adjacent injured cells.^18^, ^64^ Meanwhile, the injured cells can also form membrane protrusions, subsequently extending the adjacent stem cells to establish TNT structures. The activation of tumor suppressor molecule p53 is a necessary mechanism for the formation of TNTs, and the downstream pathway (Akt/PI3K/mTOR signaling) upregulated by p53 is also involved in nanotube formation.^65^, ^66^ Connexin 43 (Cx43) has also been demonstrated to mediate intercellular communication through TNTs.^67^ Overexpression of Cx43 facilitated mitochondrial transmission from stem cells to epithelial cells through the upregulation of tunnel tube formation.^68^, ^69^ The stress caused by rotenone or TNFα has also been shown to induce nanotube formation.^57^ The TNFα/NF‐κB/TNFαip2 pathway is upregulated in response to TNFα^70^; then, stem cells further promote the formation of TNT.^71^ Inflammation by interferon‐γ or lipopolysaccharide has also been shown to promote the expression of M‐Sec proteins associated with TNT formation.^72^, ^73^ The Rho‐GTPase Miro1 can facilitate the migration of mitochondria via TNTs between two cells. A high level of Miro1 can enhance the ability of mitochondrial transfer and the rescue potential of MSCs via TNT.^57^

### 4.2 Extracellular vesicles

Extracellular vesicles (EVs), ranging from 40 to 1000 nm, are divided into microvesicles, exosomes, and apoptotic bodies according to their size, molecular composition, and source.^39^, ^56^, ^74^, ^75^ Exosomes are small extracellular vesicles with a diameter from 30 to 150 nm.^76^, ^77^, ^78^, ^79^, ^80^ Due to the small size, exosomes are unlikely to carry intact mitochondria.^56^ Instead, they are able to transfer organelle fragments and genetic components.^56^

As larger EVs (50–1000 nm), microvesicles can contain both intact mitochondria and mtDNA.^52^, ^81^ Microvesicles are more heterogeneous structures independent of cell origin.^56^ The mechanisms of microvesicle biogenesis are associated with TSG101 protein recruitment to the cell surface.^82^ Islam et al. discovered the phenomenon of microvesicle‐mediated mitochondrial transmission from stem cells to pulmonary alveoli protects against acute lung injury.^30^ The mitochondria are packaged into vesicles containing light chain 3 (LC3) in the cytoplasm of stem cells and then integrated into outward budding in the plasma membrane.^49^ Stem cells can depolarize mitochondria to the plasma membrane via arrestin domain‐containing protein 1‐mediated microbubbles, thereby controlling intracellular oxidative stress and enhanced bioenergetics.^78^ Another study has shown that astrocytes can produce extracellular mitochondria that enter neurons to improve neuronal activity after ischemic stroke.^52^ These studies suggest that intercellular mitochondria transmission through microvesicles is an important route to rescue mitochondrial function in the damaged cells.^3^

Recently, apoptotic bodies generated from cells undergoing apoptosis have been demonstrated to be rich in mitochondria and mitochondrial components.^83^ Galleu et al. have demonstrated that the presence of labilized cytotoxic cells in patients can induce apoptosis in stem cells and predict therapeutic efficacy of stem cells.^56^, ^84^

### 4.3 Cellular fusion

Aberrant mitochondrial function and endoplasmic reticulum stress enables upregulation of cell fusion events, which might represent the adaptive mechanism that promotes cell plasticity and survival in oxidative injury.^85^ Cell fusion is a multistep process, including a cellular stress response, activation of autophagy, rearrangement of cellular cytoskeleton structure, expression of fusion protein, intercellular communication, and exchange of cytoplasm.^39^, ^85^, ^86^ It has been reported that stem cells can fuse with cardiomyocytes, airway epithelial cells, neurons, and hepatocytes.^25^, ^87^ Adrien et al. have demonstrated that stem cells reprogram myocytes into an immature state through cell fusion and mitochondrial transfer.^32^ However, the synkaryons and permanent cell fusion are scarce both in vivo and in co‐cultures, so cell fusion does not seem to be the main route of mitochondrial transfer and stem cell therapy.^32^, ^39^

### 4.4 Mitochondrial extrusion

Emerging data speculate that naked mitochondrial extrusion is another route for mitochondrial transfer. Akihito Nakajima et al. show that cytoplasmic vacuoles engulf and subsequently extrude naked mitochondria into the extracellular medium when undergoing acute TNFα‐induced apoptosis.^88^ Unuma et al. also indicate that the extrusion of mitochondria and mitochondrial contents in this process, which can provoke the inflammatory response of the immune cells.^89^ Both basophils^90^ and eosinophils^91^ can eject their mtDNA to bind and kill infectious bacteria in an ROS‐dependent manner. Boudreau et al. show that platelets can release naked mitochondria leading to inflammatory responses both in vitro and in vivo.^92^ Although the above data are supported, whether stem cells are able to release naked mitochondria into the extracellular medium as a route for therapeutic mitochondria transfer is yet to be shown.

## 5 PROTECTION MECHANISMS ON RECIPIENT CELLS

After the process of intercellular delivery, stem cell‐derived healthy mitochondria able to enter the recipient cells with defective mitochondria and corporate with the endogenous energy metabolism network.^39^ Existent data suggest that stem cell‐derived mitochondria might improve survival of recipient cells through rescuing aerobic respiration and energy metabolism, regulating mitophagy and mitochondrial biogenesis, optimizing mitochondrial dynamics, and decreasing the mtDNA mutation load (Figure 1B).

### 5.1 Rescuing aerobic respiration and energy metabolism directly

Spees et al. firstly show that the mitochondria transfer from adult stem cells can directly rescue aerobic respiration in recipient cells.^25^ It has also been demonstrated that the stem cells able to rescue cybrid cells of myoclonic epilepsy with ragged red fibers (MERRF) through providing intact mitochondria and improving mitochondrial bioenergetics.^15^ Likewise, bone marrow‐derived MSCs able to rescue energy metabolism of the cells under oxidative stress through transporting healthy mitochondria in vitro.^93^ MSCs can also transfer intact mitochondria to protect against acute central nervous system^17^ or lung injury in vivo.^30^ Therefore, it is a quick and direct way that stem cell‐derived mitochondria incorporate into the endogenous mitochondrial network to repair metabolic machinery.

### 5.2 Regulating mitochondrial biogenesis and mitophagy

For mitochondrial encephalomyopathy, stem cell‐derived healthy mitochondria are also important pathways for intracellular quality control of mitochondria. Mitophagy and biogenesis are coordinated and opposing pathways that regulate mitochondrial quality control and metabolism.^94^ Mitochondrial biogenesis is intricate process that includes transcription and translation of nuclear and mitochondrial genomes, recruitment, and import of mitochondrial proteins and lipids.^94^, ^95^, ^96^

Mitochondrial biogenesis is rigorously controlled by intracellular signaling pathways and the activation of nuclear transcription factors, such as peroxisome proliferator‐activated receptor gamma, coactivator 1 α, nuclear respiratory factors, and transcription factor A.^96^ The kinase pathways, second messenger molecules, and hormones participate in regulating the complex process.^95^, ^96^ The import of stem cell‐derived mitochondria and components may provide a quick supplement for mitochondrial biogenesis, and the exogenous mitochondria also require coordination with intracellular network in recipient cells.

Dysfunctional mitochondria are also deleterious in mitochondrial encephalomyopathy.^97^, ^98^, ^99^ Mitophagy, the selective degradation of mitochondria, is also crucial to maintain cell homeostasis. Parkin‐dependent process, which is regulated by induced putative kinase 1 (PINK1), is a well‐studied mechanism of mitophagy.^100^, ^101^, ^102^ Besides, FUN14 domain containing 1, Bcl2 interacting protein 3 (BNIP3) or BNIP3L can directly recruit autophagosomes to injured mitochondria via interaction with LC3.^100^ In addition to canonical mitochondrial degradation, mitochondrial‐derived vesicle is one important kind of mitophagy pathway.^100^, ^103^ Mitochondrial spheroid also distincts from other autophagy pathways and represents the structural remodel of mitochondria in response to oxidative stresses.^100^, ^104^, ^105^, ^106^, ^107^ The transmitophagy has also been observed in stem cells that mitochondria released from damaged cells are engulfed by stem cells,^108^, ^109^ then stem cells can degrade the damaged mitochondria and produce healthy mitochondria against programmed cell death.^42^ Except the mitochondrial transfer, the transport of progenitor cell‐derived lysosomes via TNT is also observed and involved in the autophagy process of stressed cells.^110^ Therefore, stem cells may involve in regulating both intra‐ and intercellular mitophagy and biogenesis process of mitochondria in host cells with mitochondrial disorders.

### 5.3 Optimizing mitochondrial dynamics

As high dynamic organelles, mitochondria fission and fusion are crucial for quality control. The fission can segregate dysfunctional mitochondria, while fusion can share healthy mitochondrial components.^100^, ^111^, ^112^ Mitochondrial encephalomyopathies always have the imbalance of mitochondrial dynamics in skeletal muscle or nervous system.^113^ It has the possibility that stem cell‐derived mitochondria may involve in the mitochondria dynamics in recipient cells of mitochondrial encephalomyopathies, especially the fission and fusion process.^108^ A 3D imaging of the cells shows that the MSC mitochondria are spread out throughout the endogenous recipient mitochondria network.^114^

Mitochondrial fusion is controlled by three dynamin‐related GTPase proteins. The fusion of the outer mitochondrial membrane is regulated by mitofusin 1 (Mfn1) and Mfn2,^103^, ^115^, ^116^ and the fusion of the inner mitochondrial membrane is regulated by optic atrophy 1 (OPA1).^103^, ^117^, ^118^ Recently, another study demonstrates that stem cells play protective role in a mitochondrial dysfunction mice model through the mechanisms of transferring mitochondria and increasing the fusion gene expression of OPA1, Mfn1, and Mfn2 in host cells.^119^

Mitochondrial fission is mediated by the dynamin‐related protein 1 (Drp 1), which binds to four receptors: fission 1 protein (Fis1), mitochondria fission factor, mitochondrial dynamics protein of 49 and 51 kDa.^100^, ^103^, ^120^ Chuang et al. show that the stem cells able to rescue MERRF cybrid cells and optimize mitochondrial dynamics through donating healthy mitochondria.^15^ The MERRF cybrid cells present decreased OPA1 and increased Fis1, which cause the imbalance of mitochondrial fusion and fission.^15^ It is demonstrated that stem cells can recapture the dysfunctional mitochondria network and abnormal fusion/fission protein in MERRF cybrid cells through mitochondria transfer.^15^

### 5.4 Decreasing the mtDNA mutation load

Majority of mitochondrial encephalomyopathies is caused by the pathogenic mtDNA mutations. Previous research find that the level of the mtDNA mutations will also influence disease severity and is reliable measure for clinical assessment.^121^, ^122^ Therefore, reducing mutation load through intercellular exchange of mtDNA may have a certain effect on alleviating clinical severity of mitochondrial encephalomyopathy. The transfer of low copy number of mtDNA can recover the mitochondrial function of immunoincompetent mice.^123^ Jayaprakash et al. find the horizontal transfer of mitochondrial DNA between different cell lines in the co‐cultures.^124^ Tumor cells can repair the transcription and translation of mtDNA and improve mitochondrial bioenergetics through acquiring healthy mtDNA from ambient cells.^39^ It is also proposed that stem cells can partly reduce mtDNA mutation load via providing healthy mtDNA and mitochondria, which is sufficient to recover the mitochondrial respiration in MERRF cybrid cells long term.^15^ The routes of mtDNA transfer not only restrict to TNTs, microvesicles, cellular fusion, and mitochondrial extrusion,^125^, ^126^, ^127^ but also including more tiny structures such as exosomes and gap junction.^127^, ^128^ However, emerging data challenge the potential therapeutic use of EV‐based delivery systems for mtDNA‐based diseases.^129^ After exposure to the donor mtDNA in EV fractions for years, there is little transfer of the donor mtDNA to the host mtDNA fraction in subjects tissues.^129^

## 6 FUTURE PERSPECTIVES AND CHALLENGES

An increasing number of diseases are being found to have the pathogenesis of mitochondrial dysfunction.^130^ However, the treatments for mitochondrial disorders are inadequate, especially in the therapies of mitochondrial encephalomyopathies. The main strategy employed in the treatment of nDNA‐based mitochondrial encephalomyopathy, while current capacity to repair or replace mDNA in somatic cells is inadequate. Stem cells have the characteristic of lower immunogenicity and the ability for long‐term proliferation to amplify the quantity of mitochondria.^49^ Thus, stem cells are an ideal choice as potential mitochondrial donors.

The transplantation of stem cell‐derived mitochondria to somatic cells has been demonstrated not only in vitro but also in vivo and is recognized as a novel and promising strategy for treating mitochondrial dysfunction. However, there are still several major challenges and concerns remaining. Time, number, efficiency, and route of mitochondrial transfer are important for the activity restoration of the recipient cells bearing mitochondria dysfunction.^39^, ^72^, ^73^ Various transfer routes of stem cell‐derived mitochondria include TNTs, extracellular microvesicles, cellular fusion, and mitochondrial extrusion, which have significant differences in the dosage and efficiency of mitochondria transfer and may directly affect the rescue effect of stem cells.^39^, ^72^, ^73^ TNTs are known as the highway for intercellular organelle transport,^54^ which is much more efficient than other modes of intercellular mitochondria delivery, such as the extracellular microvesicles and mitochondrial extrusion.^43^ In spite of this, it is possible that different transfer routes might complement each other in different pathophysiological stages and microenvironments, and even cooperate with each other to promote the therapeutic effect of stem cells. On the other hand, various mitochondria transfer routes have respective signaling pathways. The identification of these signaling pathways and the mechanisms of intercellular mitochondria delivery will improve the potential applications of cell therapy‐based mitochondrial restoration.^131^, ^132^

Future therapeutic investigations should consider strategies to pharmacologically enhance or control the transfer of stem cell‐derived mitochondria,^39^ especially in stem cell therapy for mitochondrial encephalomyopathy.

Moreover, the mechanisms through which stem cell‐derived mitochondria can be incorporated into the endogenous energy metabolism network remain to be elucidated.^39^

Furthermore, mitochondrial damage and ROS are considered to be probably involved in the inflammation.^133^ The mitochondrial dysfunction could also play an important role in chronic inflammation of the neurodegenerative disorders and mitochondrial diseases.^134^ The transplantation of stem cell‐derived mitochondria may be used as an effective treatment for these pathologies, which may attenuate production of ROS and have immunomodulatory effects.^133^, ^134^

Future research on the molecular mechanisms underlying the improvement of aerobic respiration, dynamics, and the quality control of transplanted mitochondria will accelerate the development of stem cell treatment in mitochondrial encephalomyopathies. However, most current studies on mitochondrial disease remain in the in vitro stage due to a lack of mtDNA‐based animal models of mitochondrial encephalomyopathies. Meanwhile, novel tracking technologies are necessary to unravel the mechanism of intercellular mitochondria transmission in vivo. The development of cell heteroplasmy in single cells and mitoception to detect mitochondrial transfer^114^ are useful to unambiguously assess mitochondria transmission and the effects of stem cell mitochondria on cell metabolism and function studies.^39^

## 7 CONCLUSION

Currently, the treatment of mitochondrial encephalomyopathy faces serious challenges.^39^ Restoring the function of mitochondria and rescuing damaged mitochondria are crucial for treating mitochondrial disorders. Stem cell‐derived mitochondria transplantation has been demonstrated to play a significant role not only in metabolic rescue but also in mitochondrial dynamics, quality control, and reduction of mutation load, which may eventually prevent cell apoptosis. Thus, the therapy offers great promise for mitochondrial encephalomyopathies. Meanwhile, mitochondrial integrity and mitochondrial dynamics also become dysfunctional during some neurodegenerative diseases.^135^, ^136^, ^137^, ^138^The application of stem cell‐derived mitochondria transplantation has also attracted attention for its potential to treat many diseases with the pathogenesis of mitochondrial dysfunction, such as cerebral vascular disease,^139^, ^140^ Parkinson's disease, dementia, amyotrophic lateral sclerosis, myocardial ischemia–reperfusion injury, and acute lung injury.^30^, ^71^, ^74^, ^108^, ^141^, ^142^, ^143^, ^144^ However, as one of the most representative and intractable mitochondrial diseases, mitochondrial encephalomyopathies should be the first to see a breakthrough and benefit from this novel treatment strategy.

## CONFLICT OF INTEREST

The authors declare no conflict of interest.

## Data Availability

Data sharing not applicable to this article as no datasets were generated or analyzed during the current study.
